# Antioxidant Activities of Pentacyclic Triterpenes From *Melipona beecheii* Propolis and Their Semisynthetic Derivatives

**DOI:** 10.1002/cbdv.71689

**Published:** 2026-09-10

**Authors:** Aarón Santana‐Hernández, Alejandro Yam‐Puc, Pamela Yah‐Nahuat, María Vargas‐Vargas, Rocío Borges‐Argáez, Julio Aguiar‐Pech, Jesús Ramón‐Sierra, Elizabeth Ortiz‐Vázquez

**Affiliations:** ^1^ División de Estudios de Posgrado e Investigación Tecnológico Nacional de México/ ITMérida Mérida Yucatán México; ^2^ Unidad de Biotecnología Mérida Yucatán México

**Keywords:** antioxidant activity, *Melipona beecheii*, pentacyclic triterpenes, propolis, stingless bees

## Abstract

Four principal fractions (**I**‐**IV**) were obtained from the *Melipona beecheii* propolis. GC‐MS analysis of these fractions allowed us to recognize 11 pentacyclic triterpenes (PTs), fraction **I** [β‐amyrin acetate (**1**), germanicol acetate (**2**), moretenol acetate (**3**) and 24‐methylencycloartan‐3‐ol acetate (**4**)]; fraction **II** [marsformosanone (**5**), β‐amyrenone (**6**), germanicone (**7**) and lupenone (**8**)]; fraction **III** [β‐amyrin (**9**) and, glutinol (**10**)]; and fraction **IV** [**9** and α‐amyrin (**11**)]. This is the first time that triterpenes **7** and **10** are reported in the propolis of *M. beecheii*. Three semisynthetic derivatives were prepared: the reduction of fraction **II** and the acetylation and oxidation of fraction **IV**. The ethanolic extract of propolis and fraction **III** presented the highest free DPPH radical scavenging capacity at 53.89 ± 8.39% and 47.77 ± 0.91%, respectively, using a concentration of 10 mg/mL. In contrast, the reducing power of Fe (III) analysis showed a strong reducing power of Fe(III) for all fractions and their derivatives with an EC_50_ = 0.8–1.0 mg/mL.

## Graphical abstract

*M. beecheii* propolis contains eleven identified PTs, including germanicone and glutinol, reported for the first time in this material. Antioxidant assays reveal Fe(III)‐reducing activity at an effective concentrations lower than DPPH radical‐scavenging activity. C‐3 derivatization alters responses in triterpene‐rich fractions, supporting a preliminary relationship between C‐3 functionality and their in vitro redox behavior.

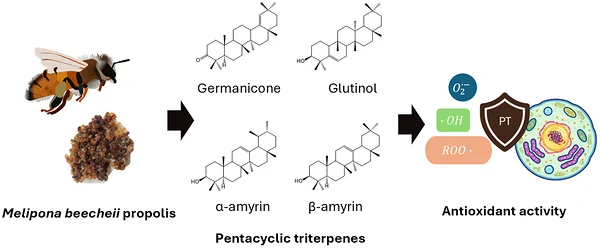

## 1 Introduction

Oxidative stress results from an imbalance between reactive oxygen species (ROS) and endogenous antioxidant defenses. It is implicated in chronic inflammation, metabolic disorders, neurodegeneration, and infectious diseases [1]. Therefore, exogenous antioxidants from natural sources have gained interest because of their potential health benefits. They may support the development of new strategies against oxidative stress‐related diseases. They also provide useful models for understanding biochemical defense mechanisms [2].

Bee products, including honey, pollen and propolis, are important natural sources of antioxidant molecules. They are also associated with agro‐industrial innovation and sustainable production practices [3]. Stingless bee propolis has attracted attention as a value‐added product from tropical regions. This interest is supported by studies describing its biological and antioxidant properties [4, 5].

*Melipona beecheii* is a stingless bee widely distributed in Central America and Southern Mexico. Its propolis has several reported biological properties. However, its antioxidant potential has not been rigorously characterized compared with other stingless bee propolis [5]. Few studies have linked its antioxidant capacity with its chemical profile. This profile has been described as rich in pentacyclic triterpenes (PTs) and phenolic compounds [6, 7].

PTs are prominent constituents of the lipophilic fraction of *M. beecheii* propolis. These compounds include ursane, oleanane, friedelane and lupane PTs, such as α‐ and β‐ amyrin and lupeol) [8, 9]. PTs are relevant because they can modulate physiological processes associated with oxidative stress [10, 11]. They represent one of the most widespread groups of secondary metabolites. Their biological properties include antioxidant, anti‐inflammatory, and antimicrobial effects [12, 13]. Chemical derivatization studies also reflect the versatility and pharmacological relevance of this metabolite family.

This study evaluated the eleven main PTs from the lipophilic fraction of *M. beecheii* propolis. It also evaluated their semisynthetic derivatives. The aim was to determine how chemical modification influenced their antioxidant activity in vitro.

## 2 Results and Discussion

### 2.1 Pentacyclic Triterpenes: Identification and Derivatization

**FIGURE 1:**
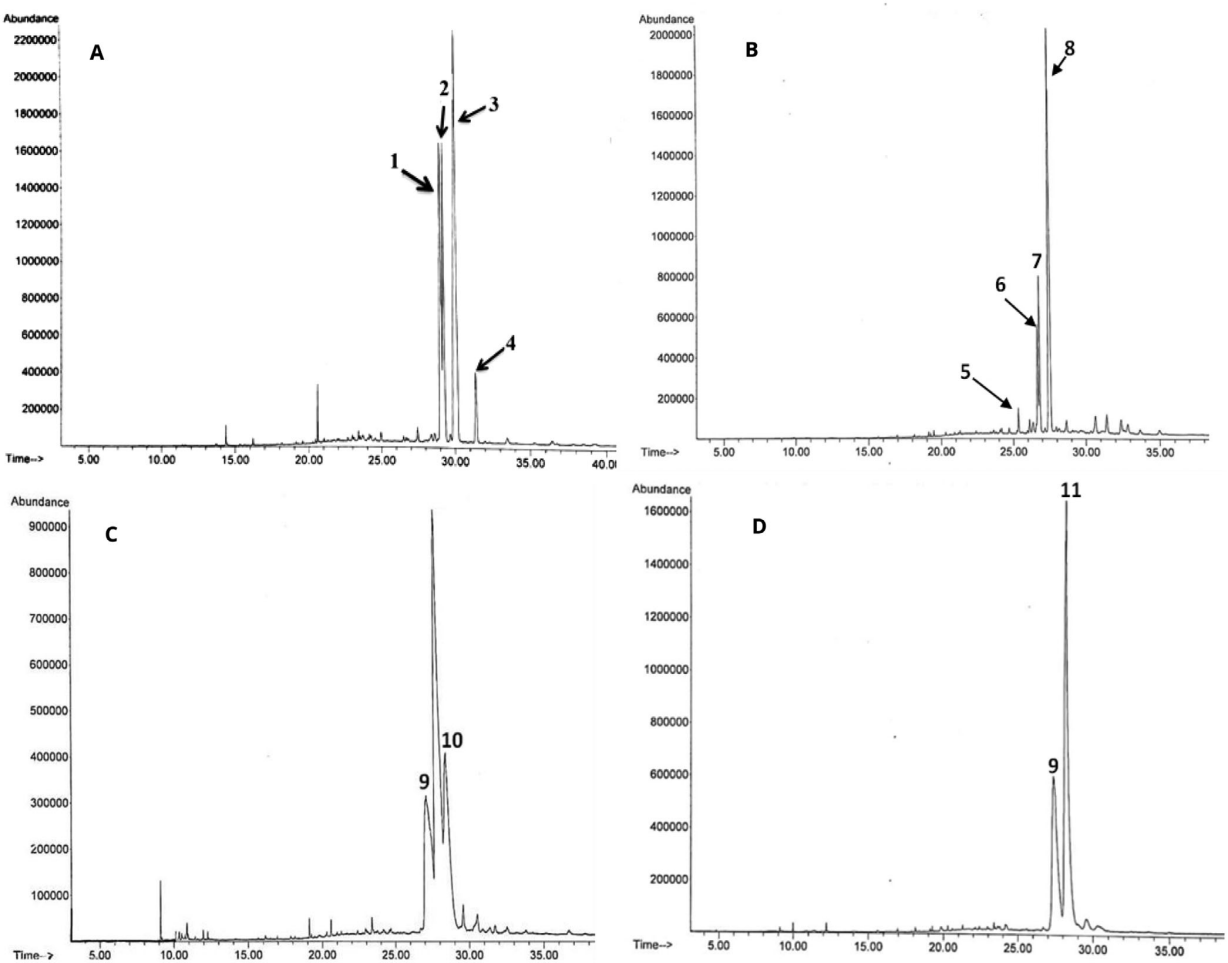
GC‐MS profile of fractions **I–IV** of *M. beecheii* propolis. (A) Fraction **I**, (B) Fraction **II**, (C) Fraction **III,** and (D) Fraction **IV**.

The ethanolic extract of *M. beecheii* propolis (EEP) was successfully fractionated with hexane. Further purification of the hexane/ethyl ether partitions by column chromatography yielded four main fractions (**I**‐**IV**). GC‐MS analysis (Figure 1) allowed the identification of PTs mixture in each fraction. These fractions were grouped according to the functional group at C‐3: acetylated compounds in fraction **I**, ketones in fraction **II** and hydroxylated compounds in fractions **III** and **IV**.

Eleven PTs were identified in the four fractions. Fraction **I** contained [β‐amyrin acetate (**1**), germanicol acetate (**2**), moretenol acetate (**3**), and 24‐methylencycloartan‐3‐ol acetate (**4**)]; fraction **II** contained [marsformosanone (**5**), β‐amyrenone (**6**), germanicone (**7**), and lupenone (**8**)]; fraction **III** contained [β‐amyrin (**9**) and glutinol (**10**)]; and fraction **IV** contained [**9** and α‐amyrin (**11**)].

The metabolites were identified based on their molecular weights and mass spectral fragmentation patterns. These data were compared with those available in the in the NIST05 mass spectral library. Yam Puc et al. (2019) [8] previously reported the PTs **1**‐**6**, **8, 9,** and **11** in *M. beecheii* propolis (Figures S1–S6, S8, S9 and S11). In the present study, PTs **7** and **10** are reported for the first time in a propolis sample of that species. Fractions **II** and **IV** were derivatized, whereas fractions **I** and **III** were not.

**FIGURE 2:**
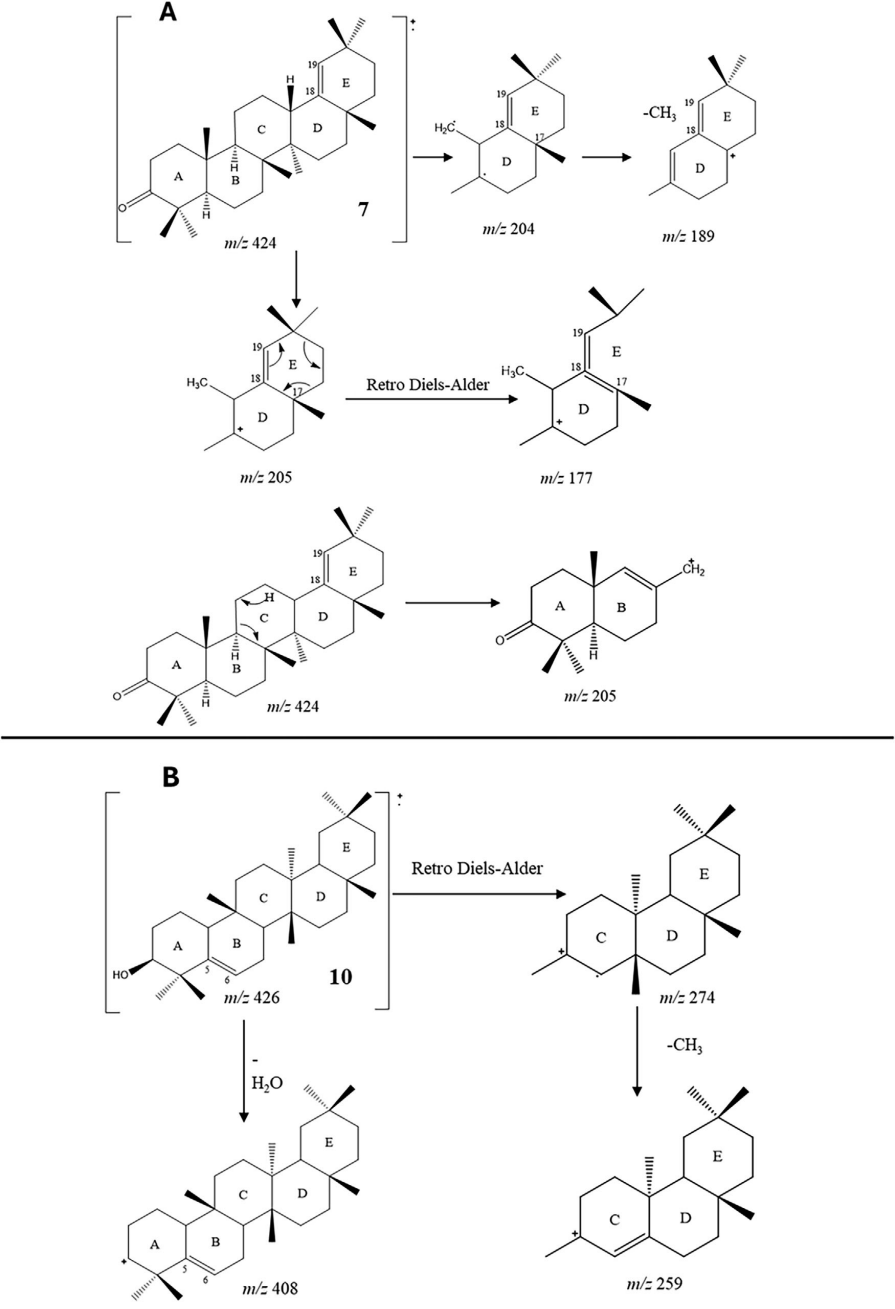
Mass spectral fragmentation patterns of metabolites (A) germanicone (**7**) and (B) glutinol (**10**).

GC‐MS analysis of fraction **II** allowed the identification of metabolite **7** (t_R_ 26.851 min). This compound showed a molecular ion at m/z 424, suggesting the molecular formula C_30_H_48_O (Figure S7). It was identified as germanicone, an ^18^Δ oleanane‐type PT and an oxidation product of germanicol. The assignment was supported by the fragment ions at m/z 409 (M^+^‐Me), 205, 204, 189, and 177. The ion at m/z 205 was attributed to hydrogen transfer from rings D and E to rings A and B. The ion at m/z 204 was assigned to rings D and E, whereas the ion at m/z 189 correspond to 204‐Me. The fragment at 177 was associated with a retro‐Diels‐Alder rearrangement involving rings C and D. This rearrangement is related to the unsaturation between C‐18 and C‐19 (Figure 2A) [14, 15]. Germanicone has been identified in propolis from the Republic of Cameroon, Africa. It has also been identified in yellow propolis from Mato Grosso, Brazil, and in the chloroform extract of *A. mellifera* propolis from São Paulo, Brazil [16, 17, 18].

GC‐MS analysis of fraction **III** allowed identification of **10** (t_R_ 28.496 min). The assignment was supported by fragment ions at m/z 426, suggesting the molecular formula C_30_H_50_O (Figure S10). Its structure includes a double bond between carbons C‐5 and C‐6 and a secondary alcohol at C‐3. The compound was identified as glutinol. This assignment was supported by the fragment ions at m/z 408 (M^+^‐H_2_O), 411 (M^+^‐CH_3_), 393 (411‐ H_2_O), 274, 259, 205, 134, and 95 [19, 20]. The main fragmentation in this molecule involves the retro‐Diels‐Alder rearrangement, which occurs in ring B. This rearrangement occurs due to the double ring between carbons C‐5 and C‐6. It generates an intense peak at m/z 274. Loss of a methyl group produces the base peak at m/z 259 (Figure 2B) [21, 22, 23, 24]. Glutinol has been identified in Brazilian red propolis. It has also been reported in extracts from species of the genus Eugenia. This genus is known for its diverse medicinal properties, including anti‐inflammatory, hypoglycemic, diuretic, and analgesic effects. It has also been used in traditional treatments for stomach disorders [25, 26].

**FIGURE 3:**
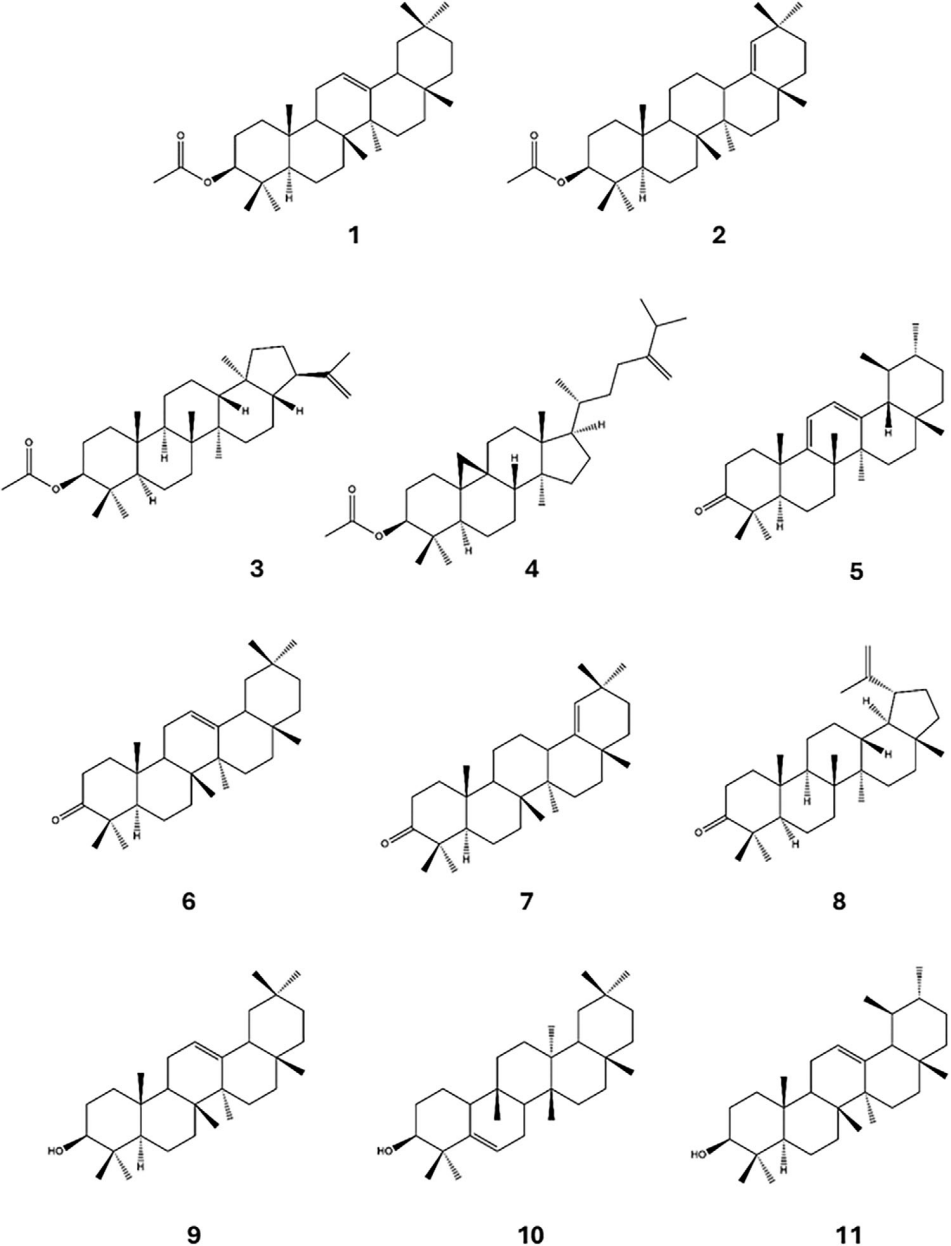
Structures of Pentacyclic Triterpenes (PTs) identified from *M. beecheii* propolis.

^1^H‐NMR analysis of the four fractions allowed the identification of the 11 PTs (Figure 3 and Table 1). The presence of acetylated PTs (**1**‐**4**) was observed in fraction **I**. These compounds showed a proton attached to the oxygenated carbon (H‐3) at δ 4.48 (m). They also showed the acetate methyl signal at δ 2.04 (s). The presence of β‐amyrin acetate (**1**) was supported by the vinylic proton H‐12 at δ 5.18 (t, J = 3.5 Hz). Germanicol acetate (**2**) was identified by the vinylic proton H‐19 at δ 4.86 (s) [27]. Moretenol acetate (3) was identified by two singlets at δ 4.68 (H‐29a) and 4.56 (H‐29b). This signals correspond to the nonequivalent vinylic protons of the methylene group H‐29. The signal at δ 1.68 (s) corresponded to the allylic methyl protons H‐30 [28, 29]. Furthermore, the presence of 24‐methylencicloartan‐3‐ol acetate (**4**) was revealed by the signals for protons at δ 0.57 (H‐29a, d, J = 4.0 Hz) and 0.35 (H‐ 29b, d, J = 4.0 Hz) indicating a cycloartane skeleton (Figure S12A) [30, 31].

**TABLE 1:** PTs identified from *M. beecheii* propolis.

Fraction	Metabolite number	t*_R_* (min)	Metabolite	Molecular formula	Molecular weight
**I**	**1**	29.11	β‐amyrin acetate	C_32_H_52_O_2_	468
	**2**	29.34	Germanicol acetate	C_32_H_52_O_2_	468
	**3**	30.20	Moretenol acetate	C_32_H_52_O_2_	468
	**4**	31.43	24‐methylencycloartan‐3‐ol acetate	C_33_H_54_O_2_	482
**II**	**5**	25.35	Marsformosanone	C_30_ H_46_O	422
	**6**	26.72	β‐amyrenone	C_30_H_48_O	424
	**7**	26.851	Germanicone	C_30_H_48_O	424
	**8**	27.58	Lupenone	C_30_H_48_O	424
**III**	**9**	27.14	β‐amyrin	C_30_H_50_O	426
	**10**	28.49	Glutinol	C_30_H_50_O	426
**IV**	**9**	27.36	β‐amyrin	C_30_H_50_O	426
	**11**	28.15	α‐amyrin	C_30_H_50_O	426

Fraction **III** showed characteristic signals for PTs **9** and **10**. A multiplet at δ 3.38 was assigned to the H‐3 proton. A triplet at δ 5.18 (J = 3.6 Hz) was assigned to the vinylic proton H‐12. Traces of α‐amyrin (**11**) were detected from the vinylic proton at δ 5.13 (t, J = 3.6 Hz [32]. Characteristic signals for glutinol (**10**) included the proton H‐3 at δ 3.46 (br s). The vinylic proton H‐6 appeared at δ 5.63 (d, J = 6.0 Hz) (Figure S12B) [26].

Fraction **II** and its reduction products were confirmed by chemical displacements of protons at C‐3. The PTs present in this fraction were converted into the corresponding secondary alcohols. As mentioned above, GC‐MS analysis of fraction **II**. However, the corresponding peak of marsformosanone (**5**) was of very small size (Figure 1B). Because of this, the ^1^H‐NMR analysis of fraction **II** indicated the presence of PTs **6**‐**8**.

**FIGURE 4:**
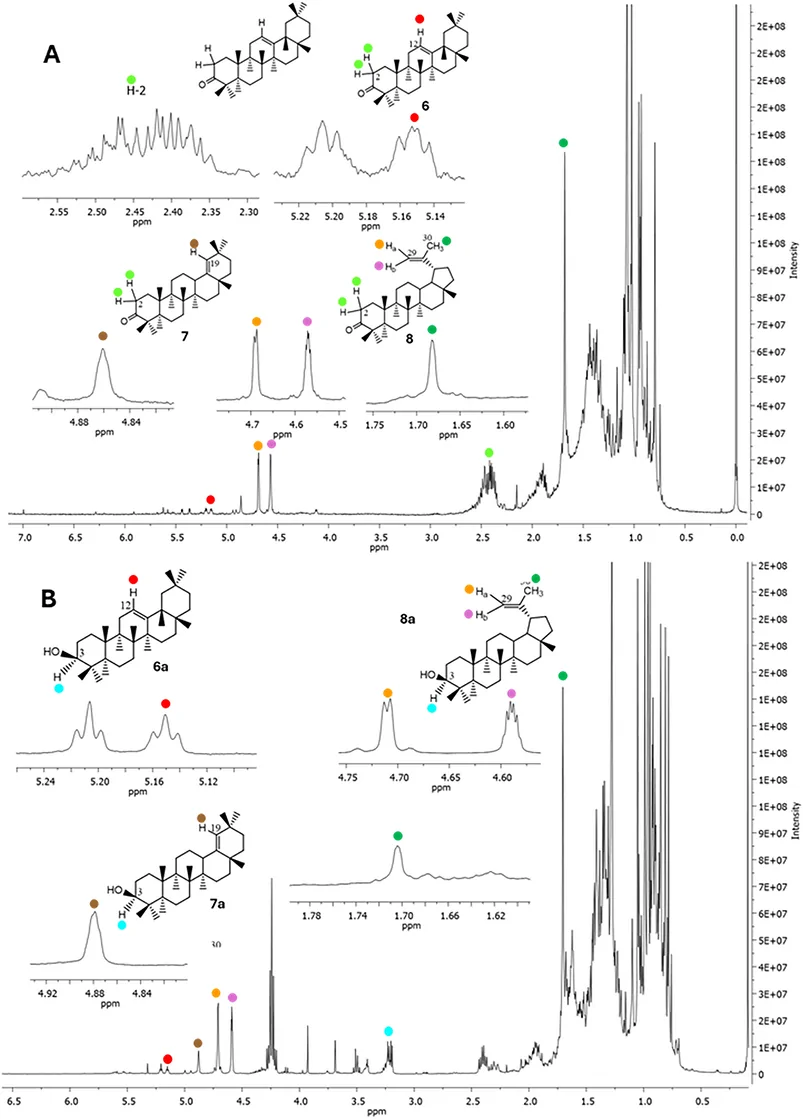
^1^H‐NMR spectra of PTs present in fraction **II**: (A) β‐amyrenone (**6**), germanicone (**7**), lupenone (**8**); and their reduced derivatives (B) **6a**, **7a,** and **8a** from *M. beecheii* propolis.

A signal at δ 2.42 (m) was assigned to protons ă to the ketone group, H‐2 (Figure 4A). β‐amyrenone (**6**) was identified by the signal for the vinylic proton H‐12 at δ 5.15 (t, J = 3.5 Hz). Germanicone (**7**) was supported by the vinylic proton H‐19 at δ 4.86 (s) [33, 34, 35, 36]. Lupenone (**8**) was corroborated by vinylic proton signals at δ 4.69 (H‐29a, d, J = 2.3 Hz) and 4.57 (H‐29b, dd J = 1.4, 2.4 Hz). The singlet at δ 1.68 corresponded to the allylic methyl group H‐30 [35, 37]. After reduction of fraction **II** with NaBH_4_, a new signal appeared at δ 3.23 (m). This signal corresponded to the proton H‐3 of the newly formed secondary alcohol (Figures 4B and S14).

The presence of α‐amyrin (**11**) and β‐amyrin (**9**) in fraction **IV** was confirmed by ^1^H‐NMR. Both PTs showed the protons H‐3 at δ 3.20 (m). The diagnostic signals for PTs **11** and **9** corresponded to the vinylic proton H‐12. This proton shows distinctive chemical shifts for both PTs, as reported in the literature [32, 38]. For α‐amyrin (**11**), H‐12 appeared at δ 5.13 (t, J = 3.1 Hz). For β‐amyrin (9), H‐12 was detected at δ 5.18 (t, J = 3.1 Hz) (Figures S13 and S15A) [32].

**FIGURE 5:**
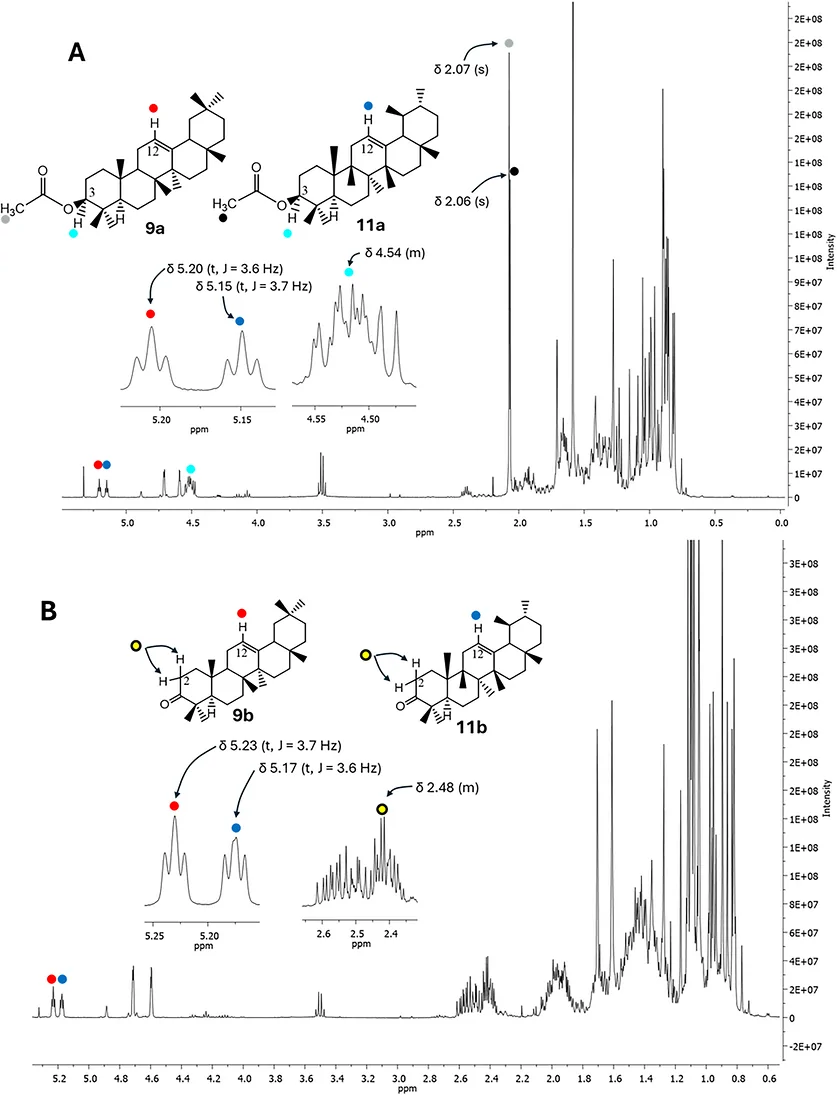
^1^H‐NMR characteristic chemical displacements of PTs corresponding to fraction **Iva**: (A) β‐amyrin acetate (**9a**) and α‐amyrin acetate (**11a**) and, fraction **IVb**: (B) β‐amyrenone (**9a**) and α‐amyrenone (**11a**) from *M. beecheii* propolis.

Acetylation of fraction **IV** to obtain fraction **IVa** was confirmed by ^1^H‐NMR. The acetate methyl residues appeared at δ 2.06 (s) and 2.07 (s). In the acetylated derivative, H‐3 shifted downfield from δ 3.20 (m) and 4.54 (m) (Figures 5A and S15B) [39]. Treatment of fraction **IV** with PCC produced the oxidized derivative **IVb**. Oxidation was confirmed in ^1^H‐NMR. The signal for proton H‐3 at δ 3.20 (m). A new signal appeared at δ 2.48 (m), typical of α‐protons adjacent to a ketone (Figures 5B and S15C) [33, 35, 40].

The present study has some limitations that should be acknowledged. Antioxidant activity was assessed only through DPPH radical‐scavenging and Fe(III)‐reducing power assays. No cellular or biological models were used to evaluate the antioxidant effects of the identified metabolites or their derivatives. Therefore, these findings should be interpreted as in vitro chemical evidence and should not be directly extrapolated to in vivo effects.

### 2.2 Antioxidant Activity

**FIGURE 6:**
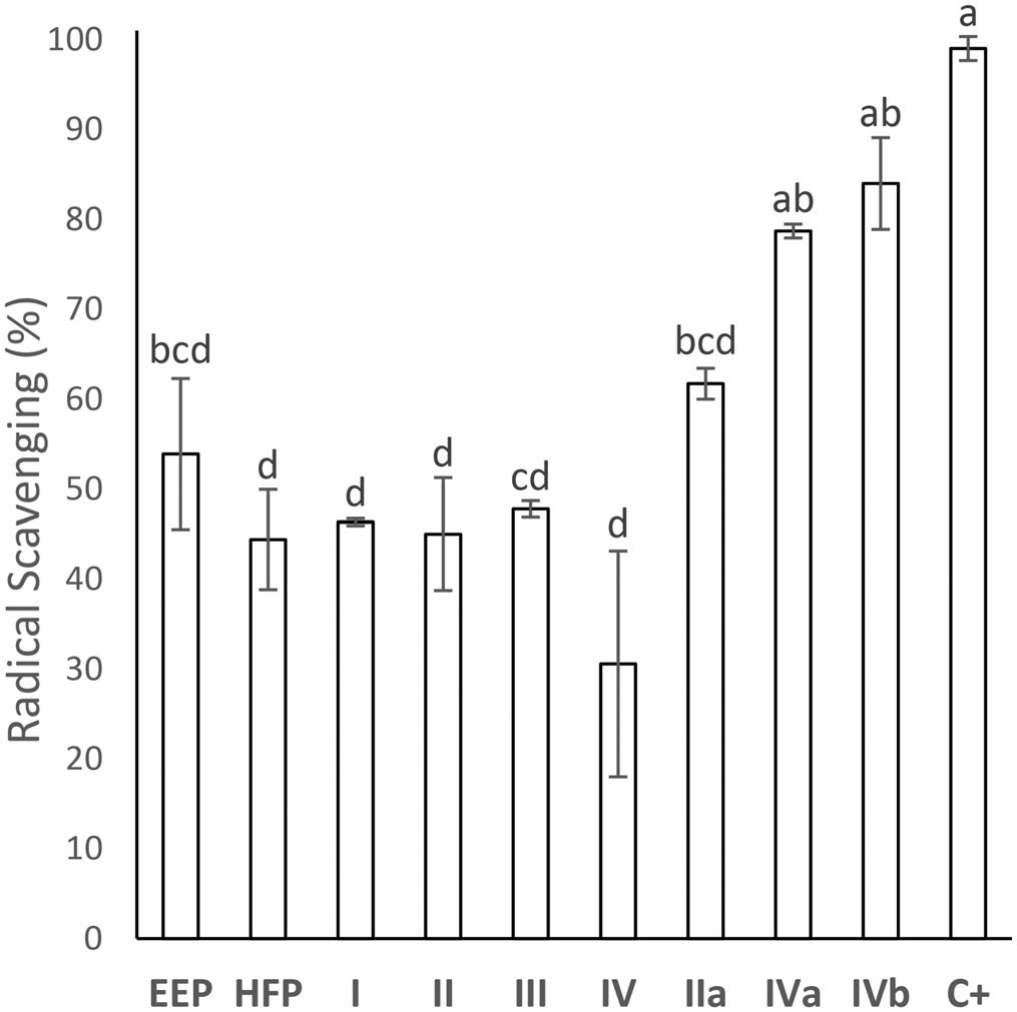
Percentage of free DPPH radical‐scavenging activity at 10 mg/mL of ethanolic extract of propolis (EEP), Hexane Fraction from Propolis (HFP) and the fractions rich in PTs from *M. beecheii* (**I**‐**IV**) and their reduced (**IIa**), acetylated (**IVa**) and oxidized (**IVb**) derivatives. Values are expressed as mean ± standard deviation (n = 3). Different letters indicate significant differences among samples (α = 0.05).

DPPH radical‐scavenging activities of tested samples are summarized in Figure 6 and Table 2. EEP and fraction **III** showed the highest in vitro antioxidant capacities. The oxidized derivative of fraction **IV** showed an even stronger DPPH scavenging efficiency (83.97% ± 1.0%) at a concentration of 10 mg/mL, with an EC_50_ of 0.9 mg/mL. These results suggest that chemical modifications can markedly alter the redox behavior of PTs obtained in this study. Such modifications may also enhance their potential as radical‐scavenging agents. In this case, the ketone function at C‐3 in the PTs of fraction **IVb** improved DPPH radical‐scavenging efficiency.

**TABLE 2:** Reducing Power of Fe(III) and DPPH Half Maximal Effective Concentration of EEP, HFP, the fractions rich in PTs (**I**–**IV)** and their derivatives (**IIa**, **IVa** and **IVb**).

Fraction	EC_50_ Fe(III)‐reducing antioxidant power (mg/mL)	EC_50_ DPPH (mg/mL)
EEP	0.08	10.0
HFP	0.1	15.0
**I**	0.18	13.0
**II**	0.9	15.0
**III**	0.2	18.0
**IV**	1	25.0
**IIa**	0.18	9.0
**IVa**	0.1	12.0
**IVb**	0.3	9.0
**Ascorbic acid**	0.01	0.025

The Fe(III)‐reducing antioxidant power and DPPH radical‐scavenging activity of PTs and their derivatives are summarized in Table 2. EEP, HFP, and the acetylated derivative of fraction **IV** showed the lowest EC_50_ values. Overall, Fe(III)‐reducing antioxidant power was stronger than the obtained in the DPPH assay.

The present study suggests that *M. beecheii* propolis is an important source of PTs. Its in vitro antioxidant activity is related to structural variation at C‐3. Previous studies indicate that C‐3 modification can affect the physicochemical and biological properties of PTs. These modifications include hydroxylation, acetylation formylation, and oxidation [41, 42, 43]. Therefore, C‐3‐oriented semisynthesis of PTs from the propolis of this bee species may help explain their antioxidant behavior.

The weak radical‐scavenging capacity observed in DPPH contrasts with the strong Fe(III)‐reducing power. This was especially evident in PT‐rich fractions with EC_50_ Fe(III)‐reducing power values of 0.8–1.0 mg/mL. These findings suggest that antioxidant activity is mainly associated with electron‐transfer processes. In contrast, efficient hydrogen atom‐transfer activity is less relevant in these fractions [44, 45, 46].

This distinction is important because antioxidant tests should not be interpreted interchangeably. DPPH tests can involve mixed hydrogen atom‐transfer and electron‐transfer mechanisms [47, 48]. In contrast, the Fe(III)‐reducing power test mainly evaluates reducing capacity through electron transfer [44, 45, 49].

Although PTs are mainly aliphatic molecules, their functional groups can modify their antioxidant behavior. Hydroxyl, carbonyl, and acetyl ester groups may affect polarity, hydrogen‐bonding capacity, and α‐C‐H bond reactivity. These functional groups were observed in the compounds identified in this study. According to organic oxidation principles described for alcohols and ketones, such variations can affect radical‐neutralization pathways. These pathways may involve hydrogen atom transfer, electron transfer, or both mechanisms [50].

In this context, the predominance of 3‐oxo PTs in fraction **II** is particularly relevant. Although they lack phenolic hydroxyl groups, they may still contribute to antioxidant activity in vitro and should not be considered primary radical scavengers. C‐3 carbonyl group may modify the electronic distribution of the triterpene skeleton. It may also influence the physicochemical properties of these molecules [47, 51]. These structural features could contribute to Fe(III)‐reducing power. They may also participate, together with other extract constituents, in DPPH radical‐scavenging activity [6, 44].

The results obtained after chemical modification of some fractions support this interpretation. For example, derivatization of fraction **II** reduced the EC_50_ of 3‐oxo PTs from 0.9 to 0.18 mg/mL. This suggests that converting carbonyl groups into hydroxyl groups may enhance radical‐scavenging capacity [52, 53]. Conversely, the oxidized derivative of fraction IV showed the highest DPPH activity. This indicates that oxidation can also favorably modify the in vitro antioxidant response. However, this effect likely depends on the triterpenoid skeleton and the corresponding functional group. Similar structure‐dependent effects have been reported for other triterpene derivatives [51, 54].

Overall, these results suggest that the radical‐scavenging activity of *M. beecheii* propolis is partly related to PTs. This activity may also depend on the functional groups present at C‐3. However, the identified PTs were not isolated or evaluated individually. Therefore, this interpretation should be considered a tentative structure–activity relationship rather than a definitive mechanism.

## 3 Conclusions

This study characterized the PTs profile of *M. beecheii* propolis from Yucatan. It also evaluated its in vitro antioxidant activity before and after chemical derivatization.

Eleven PTs were identified, principally of ursane, oleanane, lupane, cycloartane, and glutinane skeletons. Nine PTs, corresponding to compounds **1**–**6**, **8**, **9**, and **11**, had been previously reported in *M. beecheii* propolis. Germanicone (7) and glutinol (10) are reported here for the first time in *M. beecheii* propolis.

This structural diversity reflects the chemotaxonomic richness of *M. beecheii* propolis from the Yucatan Peninsula. It also supports the biotechnological relevance of this stingless bee product.

The PTs were differentiated by polarity and by the functional group present at C‐3. These groups included acetate in fraction **I**, ketone in fraction **II**, and alcohol in fractions **III** and **IV**. This classification provided a preliminary structure–activity framework for interpreting the antioxidant response in vitro.

The in vitro antioxidant assays showed medium‐low DPPH radical‐scavenging activity but a higher Fe(III)‐reducing power. This effect was particularly evident for the ethanolic extract of propolis (EEP) and fraction **III**. These results suggest a predominant electron‐transfer antioxidant behavior in these samples. In contrast, hydrogen atom‐transfer activity appeared less relevant under the tested conditions.

The Fe(III)‐reducing power observed in EEP and fraction **III** supports the contribution of native chemical diversity. This diversity may influence the in vitro antioxidant behavior of *M. beecheii* propolis. This finding is relevant because few studies have directly linked this propolis antioxidant capacity in vitro with its chemical profile. Therefore, our results suggest that oxidation state, polarity, and functional groups may influence PTs redox potential.

Chemical derivatization further highlighted the relevance of C‐3 functionalization. Structural changes at this position modified the in vitro antioxidant response of PT‐rich fractions. These changes may enhance or limit antioxidant behavior in vitro, depending on the fraction or semisynthetic derivative. Thus, C‐3 functionalization appears relevant to the redox behavior of PTs from *M. beecheii* propolis.

This is the first study linking the chemical profile of *M. beecheii* propolis with its antioxidant activity. However, the identified PTs were not purified or evaluated individually. Therefore, the proposed structure–activity relationship should be considered preliminary. Future studies with purified PTs, additional antioxidant models, and biological assays are needed. These studies will help determine the specific contribution and biotechnological potential of these metabolites.

## 4 Experimental Section

### 4.1 General Procedures

Column chromatography was performed using E.M. Merck silica gel (70–230 mesh). Chromatographic bands were monitored by Thin Layer Chromatography (TLC). TLC plates were revealed by immersion in phosphomolybdic acid ceric sulphate (20 g + 2.5 g in 500 mL 5% H_2_SO_4_) solution. The TLC plates were dried and gently heated.

Gas Chromatography‐Mass Spectrometry (GC‐MS) analyses were performed in a Hewlett Packard 5890 Gas Chromatograph. The instrument was coupled to a mass detector (MSD, Model 5975). Samples were injected in Split mode under the following conditions: 1 µL; Ultra‐1 column, 25 × 0.2 mm i.d.; nitrogen at a flow rate, 1.0 mL/mL. The oven temperature program was T_1_ = 100°C (3 min), T_2 _= 280°C (30 min). The temperature gradient was 10°C/min. The injector and detector temperatures were 300°C. The components of each fraction were identified by comparing their mass spectra with reported data. Fragmentation patterns were also compared with those in the NIST05 library.

^1^H‐NMR spectra were recorded on a Bruker Advance spectrometer at 400 MHz. CDCl_3_ was used as solvent and tetramethylsilane (TMS) as intern reference.

### 4.2 *M. Beecheii* Propolis

Raw propolis (789 g) of *M. beecheii* was collected by scraping upper parts of the beehive. Samples were obtained from hives located in the “Flor de Mayo” and “u Náajil Yum Kin” meliponaries. Both meliponaries are located in Maní, Yucatan, Mexico (20.3931°N, 89.3918°W) with a separation of approximately 1.4 km between them. Samples were stored at 4°C until use.

### 4.3 Extraction and Isolation

Ground propolis was extracted three times with ethanol at room temperature for 24 h. After maceration, the dissolvent was decanted, filtered and evaporated under reduced pressure. This process yielded 15.66 g (4.67%) of ethanolic extract of propolis (EEP). The EEP was successively partitioned with hexane, yielding 6.2 g (39.6%) of the Hexane Fraction of Propolis (HFP).

The HFP was purified by column chromatography using hexane/ethyl ether as eluent. Ratios of 8:2 and 95:5 were used. This purification produced four principal fractions (**I**‐**IV**):

Fraction **I**: White powder; ^1^H‐NMR (400 MHz, CDCl_3_): δ 0.35, 0.57 [d, J = 4.0 Hz, H‐19 of 24‐methylencycloartan‐3‐ol (**4**)], δ 1.68 [s, H‐30 of lupeol acetate (**3**)], 2.04 [s, CH_3_ of acetyl groups], 4.48 (m, H‐3), 4.56 [s H‐29a of lupeol acetate (**3**)], 4.68 [s, H‐29b of lupeol acetate (**3**)], 4.86 [s, H‐19 of germanicol acetate (**2**)], 5.18 [t, J = 3.5 Hz H‐12 of β‐amyrin acetate (**1**)] TLC Rf 0.77 in hexane/ethyl ether, 8:2.
Fraction **II**: transparent oil; ^1^H‐NMR (400 MHz, CDCl_3_): δ 1.68 [s, H‐30 of lupenone (**8**)], 2.42 (m, H‐2), 4.57 [dd, H‐29b J = 1.4, 2.4 Hz of lupenone (**8**)], 4.69 [d, J = 2.3 Hz, H‐29a of lupenone (**8**)], 4.86 [s, H‐19 of germanicone (**7**)], 5.15 [s, H‐12, of β‐amyrenone (**6**)]. TLC Rf 0.68 in hexane/ethyl ether, 8:2.
Fraction **III**: brownish oil; ^1^H‐NMR (400 MHz, CDCl_3_): δ 3.38 (m, H‐3), 5.18 [t, J = 3.6 Hz, H‐12 of β‐amyrin (**9**)], 5.63 [d, J = 6.0 Hz, H‐6 of glutinol (**10**)]. TLC Rf 0.32 hexane/ethyl ether, 8:2.
Fraction **IV**: white powder; ^1^H‐NMR (400 MHz, CDCl_3_): δ 3.2 (m, H‐3), 5.13 [t, J = 3.6 Hz, α‐amyrin (**11**)], 5.18 [t, J = 3.6, H‐12 of β‐amyrin (**9**)]. TLC Rf 0.28 in hexane/ethyl ether, 8:2.

General preparation of the derivatives of PTs in fractions **II** and **IV**

Reduction of the ketonic groups at C‐3 in triterpenes From fraction **II**

Fraction **II** (41 mg) was suspended in tetrahydrofuran (5 mL). The suspension was treated with NaBH_4_ (5 eq., 207 mg) under an inert atmosphere. The mixture was stirred at room temperature for 72 h. Ethanol (20 mL) was then added. The mixture was extracted with chloroform (2, 1:1), washed with distilled water, filtered, and evaporated. Purification yielded 16.9 mg of the reduced product, Fraction **IIa**, as a white powder; For this and all derivatization reactions described below, crude reaction products were purified by silica gel column chromatography using hexane/ethyl ether (8:2).

^1^H‐NMR (400 MHz, CDCl_3_): δ 1.70 [s, H‐30 of lupeol (**8a**)], 3.23 (m, H‐3), 4.56 [dd, J = 1.4, 2.5 Hz, H‐29b of lupeol (**8a**)], 4.68 [d, J = 2.4 Hz, H‐29a of lupeol (**8a**)], 4.86 [s, H‐19 of germanicol (**7a**)] 5.15 [t, J = 3.5 Hz, H‐12 of β‐amyrin (**7a**)]; TLC Rf 0.28 in hexane/ethyl ether 8:2.

Acetylation of the hydroxyl groups at C‐3 in triterpenes From fraction **IV**

Fraction **IV** (17.4 mg) was mixed with acetic anhydride (1 mL) and pyridine (0.5 mL) and was stirred overnight at room temperature. The reaction mixture was poured into water (20 mL) and extracted three times with ethyl acetate (1:1 v/v). The organic phases were washed successively with 5% HCl and 5% NaHCO_3_, The organic phase was dried over Na_2_SO_4_ and evaporated. Purification yielded 14 mg of acetylated product as colorless needles.

^1^H‐NMR spectrum (400 MHz, CDCl_3_), δ 2.06 and 2.07 (s, CH_3_ of acetic groups), 4.52 (m, H‐3), 5.15 (t, J = 3.56 Hz, H‐12 of the β‐amyrin acetate), 5.20 (t, J = 3.66 Hz, H‐12 of the α‐amyrin acetate). TLC Rf 0.77 in hexane/ethyl ether, 8:2.

Oxidation of the hydroxyl groups at C‐3 in triterpenes From fraction **IV**

Fraction **IV** (16.9 mg) was dissolved in dichloromethane (2 mL). Pyridinium Chlorochromate (PCC, 4eq, 34.1 mg) was added. The mixture was stirred overnight, quenched with ice. It was washed with acetyl acetate and water three times. The organic phase was dried over Na_2_SO_4_ and evaporated. Purification yielded 12.5 mg of the oxidated product, fraction **IVb**, as a colorless oil;

^1^H‐NMR (400 MHz, CDCl_3_), δ 2.48 (m, H‐2), 5.17 (t, J = 3.66 Hz, H‐12 of β‐amyrin), 5.23 (t, J = 3.58 Hz, H‐12 of α‐amyrin). TLC R_f_ 0.68 in hexane/ethyl ether 8:2.

### 4.4 Antioxidant Activity

#### 4.4.1 Free Radical Scavenging Assay (DPPH)

Free radical scavenging activity was measured according to the method of Shimada et al. (1992) [55]. Each reaction contained in 0.1 mL of sample and 1 mL of a 1 mM DPPH solution in 96% ethanol. The mixture were incubated for 30 min at room temperature. Absorbance was recorded at 517 nm, and the percentage of DPPH reduction was calculated as:

$$\%\mathrm{DPPH}\;\mathrm{radical}\;\mathrm{scavenging}=\left(\frac{A_{0}-A_{1}}{A_{0}}\right)\times 100\%$$

A0 = Absorbance of the control solution
A1 = Absorbance of the samples

#### 4.4.2 Reducing power of Fe III

The Fe(III)‐reducing power test was performed as described by Oyaizu (1986) and Kumaran & Karunakaran (2006) [56, 57]. Briefly, 0.25 mL of each sample was mixed with 0.25 mL of phosphate buffer (0.2 M, pH 6.6). Then, 0.25 mL of 1% potassium ferrocyanide solution was added. The mixture was incubated at 50°C for 20 min. Then, followed by 0.25 mL of 10% trichloroacetic acid. After centrifugation at 548 × g for 10 min, 0.5 mL of supernatant was collected. The supernatant mixed with 0.4 mL of distilled water and 0.1 mL of 0.1% ferric chloride. The reaction mixture was incubated again at 50°C for 10 min. It was then cooled for 10 min, and absorbance measured at 700 nm. Reducing power (%) was calculated as follows:

$$\%\mathrm{RPRP}=\left(\frac{A_{1}-A_{0}}{A_{1}}\right)\times 100\%$$

%RP = Reductor Power Percentage
A0 = Absorbance of the control solution
A1 = Absorbance of the samples

## Author Contributions

**Aarón Santana‐Hernández**: investigation, writing – original draft, validation, methodology, formal analysis, data curation. **Jesús Ramón‐Sierra**: methodology, formal analysis. **Alejandro Yam‐Puc**: investigation, methodology, formal analysis, writing – review and editing, writing – original draft, data curation. **Rocío Borges‐Argáez**: methodology, formal analysis. **María Vargas‐Vargas**: methodology, formal analysis, writing – review and editing. **Elizabeth Ortiz‐Vázquez**: conceptualization, funding acquisition, writing – review and editing, project administration, supervision, formal analysis, resources. **Julio Aguiar‐Pech**: methodology, formal analysis. **Pamela Yah‐Nahuat**: methodology, formal analysis. All the authors have read the final manuscript and approved the submission.

## Conflicts of Interest

The authors declare no conflicts of interest.

## Supporting information




**Supporting File 1**: cbdv71689‐sup‐0001‐SuppMat.docx.

## Data Availability

The data that support the findings of this study are available on request from the corresponding author. The data are not publicly available due to privacy or ethical restrictions.
