# Supplementary material for: Antioxidant Activities of Pentacyclic Triterpenes From Melipona beecheii Propolis and Their Semisynthetic Derivatives
Source: Chem Biodivers. 2026 Sep 10;23(9):e71689. doi: 10.1002/cbdv.71689 (PMC13559864; doi:10.1002/cbdv.71689)
Supplement: Supplementary file 1 [file CBDV-23-e71689-s001.docx]

**Supplementary Material**

**Figure S1.** MS of β-amyrin acetate (**1**)

**1**

C_32_H_52_O_2_

*m/z* 468

249.0

177

204.0

**2**

C_32_H_52_O_2_

*m/z* 468

205.0

249.0

**Figure S2.** MS of germanicol acetate (**2**)

C_33_H_54_O_2_

*m/z* 482

**Figure S3.** MS of moretenol acetate (**3**)

285.0

357.0

325.0

482

**4**

**Figure S4.** MS of 24-metilencycloartan-3-ol acetate MS (**4**)

C_32_H_52_O_2_

*m/z* 468

**3**

C_30_H_46_O

*m/z* 422

283.0

269.0

**5**

**Figure S5.** MS of marsformosanone (**5**)

C_30_H_48_O

*m/z* 424

**6**

**Figure S6.** MS of β-amyrenone (**6**)

C_30_H_48_O

*m/z* 424

**7**

**Figure S7.** MS of germanicone (**7**)

C_30_H_48_O

*m/z* 424

**8**

**Figure S8.** MS of lupenone (**8**)

C_30_H_50_O

*m/z* 426

**9**

**Figure S9.** MS of β-amyrin (**9**)

C_30_H_50_O

*m/z* 426

**10**

**Figure S10.** MS of glutinol (**10**)

C_30_H_50_O

*m/z* 426

**11**

**Figure S11.** MS of α-amyrin (**11**)

**Figure S12.** ^1^H-NMR spectra and characteristic chemical displacements of the pentacyclic triterpenes fraction **I** (A): β-amyrin acetate (**1**), germanicol acetate (**2**), lupeol acetate (**4**), 24-methylen-cycloartan-3-ol acetate (**5**) and fraction **III** (B): β-amyrin (**9**) and glutinol (**10**) from *M. beecheii* propolis

**Figure S13**. ^1^H-NMR characteristic chemical displacements of PTs corresponding to fraction **IV**: β-amyrin (**9**), α-amyrin (**11**) from *M. beecheii* propolis

**Figure S14**. ^1^H-NMR Characteristic chemical displacements of PTs corresponding to fraction II: β-amyrenone (**6**), germanicone (**7**), lupenone (**8**), and their reduced derivatives (Fraction **IIa**): **6a**, **7a** and 8a from *M. beecheii* propolis.

**Figure S15**. ^1^H-NMR characteristic chemical displacements of PTs corresponding to fractions **IV** (A): β-amyrin (**9**), α-amyrin (**11**), fraction **IVa** (B): acetylated derivatives **9a**, **11a** and their oxidative derivatives and fraction **IVb** (C): **9b** and **11b**.
